# Beyond diagnostic test performance: two content-validated questionnaires assessing patient and clinician satisfaction with diagnostic tests

**DOI:** 10.1186/s41687-025-00964-4

**Published:** 2025-11-27

**Authors:** Susan N. Chang, Elizabeth Exall, Caleb Dixon, Georgina Tickler, Muhammad Mamdani, Richard Body, Louis Kuritzky, Vivian Ng, A. Joy Allen, Zune Huynh, Kate Williams

**Affiliations:** 1 Roche Molecular Systems, Inc., 4300 Hacienda Drive, Pleasanton, CA 94588 USA; 2 Acaster Lloyd Consulting Ltd., Lacon House, 84 Theobalds Road, London, WC1X 8NL UK; 3 Unity Health Toronto, 30 Bond Street, Toronto, Ontario M5B 1W8 Canada; 4 Division of Cardiovascular Science, The University of Manchester, Manchester, M13 9PL UK; 5 Emergency Department, Manchester Royal Infirmary, Manchester University NHS Foundation Trust, Manchester, M13 9WL UK; 6 Clinical Assistant Professor Emeritus, Department of Community Health and Family Medicine, University of Florida, Gainesville, FL 32608 USA; 7 Roche Diagnostics Canada, 201 Boul Armand, Frapper, Laval, QC H7V Canada; 8 Roche Diagnostics Limited, Charles Avenue, Burgess Hill, RH15 9RY UK

**Keywords:** Clinical outcome assessment, Patient-reported outcome, Instrument development, Content validation, Acute medical events, Diagnostic tests, Patient satisfaction, Clinician satisfaction, Qualitative research, Concept elicitation, Cognitive debriefing

## Abstract

**Background:**

Utilization of diagnostic tests depends not only on diagnostic accuracy but also on clinicians’ and patients’ perceptions of the diagnostic test, including ease-of-use and rapidity of results. However, there has been little focus on validated measures for diagnostic test satisfaction in the literature. Satisfaction data can provide valuable information for decision-makers evaluating whether to approve, reimburse, or implement new diagnostic tests. This study describes the development and content validation of one patient-reported outcome instrument and one clinician instrument designed to evaluate satisfaction and preferences for diagnostic tests for acute medical events.

**Methods:**

The instruments were developed using concept elicitation interviews (20 patients; 20 clinicians), clinical expert input, and cognitive debriefing interviews (10 patients; 10 clinicians). Patients (who had undergone a diagnostic test for an acute medical event within 3 months prior) and clinicians (≥2 years of experience performing diagnostic tests; 5+ tests per month) were recruited from the United States, United Kingdom, and Canada. Cognitive debriefing interviews were conducted, with evidence-based revisions made to the instruments between rounds.

**Results:**

Study findings supported the content validity of the instruments and suitability for intended use. Specifically, concept elicitation interviews revealed factors impacting patient and clinician satisfaction with diagnostic tests, informing development of the Patient Satisfaction with Diagnostic Test Questionnaire (PSDT-Q) and Clinician Satisfaction with Diagnostic Test Questionnaire (CSDT-Q). Evidence-based revisions from cognitive debriefing interviews included wording updates, response option alterations, and inclusion of additional items to facilitate conceptual comprehensiveness. The content-validated PSDT-Q comprised four sections (20 items) assessing satisfaction with the diagnostic test process, satisfaction with sample/measurement collection, emotional well-being during the diagnostic test process, and preference between two diagnostic tests (optional module). The content-validated CSDT-Q comprised three sections (12 items) assessing satisfaction, confidence, and preference for the diagnostic test under study versus standard care.

**Conclusion:**

The PSDT-Q and CSDT-Q are the first content-validated instruments to measure patient and clinician satisfaction with diagnostic tests. Ongoing clinical research will assess the psychometric properties of these instruments. Integrating evidence-based measurement tools like these into clinical studies and health technology evaluations can help ensure that the preferences of those directly impacted by diagnostic innovations are represented in decision-making.

**Supplementary Information:**

The online version contains supplementary material available at 10.1186/s41687-025-00964-4.

## Plain Language Summary

Medical diagnostic tests are used to identify diseases. Common examples include blood tests and X-rays. Traditionally, these tests are evaluated based on how accurate they are. However, the user experience is often overlooked, including factors such as how comfortable, convenient, or easy the test is. If a test is accurate but painful or difficult to interpret, patients and clinicians may not want to use it. Previously, no standard, proven questionnaires existed to measure satisfaction with these tests. To address this, we developed two new tools: the Patient Satisfaction with Diagnostic Test Questionnaire (PSDT-Q) and the Clinician Satisfaction with Diagnostic Test Questionnaire (CSDT-Q). We developed these tools following rigorous research standards for creating valid health measures. This included gathering input from patients and clinicians across the United States, United Kingdom, and Canada. This process confirmed that the tools are relevant, reliable, and scientifically valid. Measuring satisfaction helps ensure that tests address the real-world needs of the people using them, which can lead to better diagnosis and treatment. Furthermore, using these tools allows the preferences of those directly impacted by diagnostic tests to be represented in healthcare decision-making.

**Supplementary Information:**

The online version contains supplementary material available at 10.1186/s41687-025-00964-4.

## Background

Diagnostic tests are essential tools in modern medicine and can be used to determine the absence, presence, and extent of a disease or condition [1]. Diagnostic tests provide results that assist clinicians in decision-making by enabling accurate diagnoses, developing appropriate treatment plans, monitoring disease progression, and evaluating treatment effectiveness [1–3]. For example, point-of-care (POC) diagnostic tests – diagnostic tests typically conducted within the setting where the patient is seen – provide timely, precise, and real-time results that enable clinicians to diagnose and establish prompt treatment for patients during the medical visit [4, 5]. Diagnostic tests encompass a wide range of modalities. They can range from in vitro diagnostics (such as Coronavirus 2019 [COVID-19] polymerase chain reaction [PCR] tests) to simple physical examinations, laboratory analyses, imaging studies (such as X-rays, magnetic resonance imaging [MRI], computed tomography [CT] scans), and genetic testing. Diagnostic tests are estimated to inform up to 70% of clinical decisions and are recognized as a critical component of healthcare delivery and infrastructure [6, 7].

Patient and clinician satisfaction and preferences for diagnostic tests have an important historical context reflected in the literature. For example, studies show that a majority of women across diverse demographics prefer self-sampling for human papillomavirus (HPV) testing over conventional sampling methods [8, 9]. Patient and clinician satisfaction play a decisive role in preferences for glycated hemoglobin (HbA1c) POC tests for diabetes monitoring [10, 11]. Factors such as embarrassment, discomfort, invasiveness, and accessibility impact acceptability and preferences regarding diagnostic tests for colorectal screening and monitoring [12, 13]. The literature highlights patient and clinician satisfaction as pivotal in developing and successfully adopting medical testing, particularly in disease areas characterized by acute medical events [10, 11, 13–17], defined as short-term illnesses and acute exacerbations of chronic conditions [18]. For example, rapid troponin testing in the emergency department has demonstrated high levels of provider satisfaction, and providers reported that its use could encourage communication among patient care team members [17]. In a study investigating the acceptability of POC testing for sexually transmitted infections compared to standard diagnostic procedures, some patients indicated that a reduced turnaround time for results could increase satisfaction [16]. Improved patient satisfaction with diagnostic tests can enhance patient adherence to disease screening and monitoring, leading to timely diagnosis and more effective disease management [8–11, 13–15].

Health technology assessment (HTA) agencies increasingly recognize the value of data on patient and clinician satisfaction and preferences when evaluating new treatments and diagnostics [19]. Such data can provide valuable supportive information for decision-makers when evaluating whether to adopt new diagnostic tests. Engaging directly with the lived experience of individuals who have undergone or used a diagnostic test, for example, through qualitative research, can elicit data regarding satisfaction and preferences that will help support and contextualize HTA appraisals [20–22].

Clinical outcome assessment (COA) instruments, such as patient-reported outcome instruments (PROs), can be used to assess satisfaction, preferences, and values regarding health technologies, including drugs, medical devices, and diagnostic tests. While COA instruments are widely used in drug and medical device evaluations (e.g., PROs for experience with chemotherapy treatment or satisfaction with inhaler devices), no COA instruments have been published that characterize satisfaction with diagnostic tests [23–26]. Given the growing acknowledgment of lived experience evidence for diagnostics and its recent inclusion in HTA guidelines [27, 28], there is an unmet need for validated tools to standardize and measure patient and clinician satisfaction with diagnostic tests to inform future evaluation, adoption, implementation, and evolution of diagnostic testing.

Therefore, this study aimed to develop two novel COA instruments – a PRO instrument and a clinician-reported instrument – to assess patient and clinician satisfaction with diagnostic tests as an outcome. These instruments could be included in clinical studies allowing for patient and clinician satisfaction to be measured alongside other outcomes, such as time-to-therapy, appropriateness of treatment, and hospitalizations. The instruments were designed to be suitable for assessing various diagnostic evaluations, including POC tests, conventional diagnostics (e.g., laboratory tests) and scenarios where diagnosis relies solely on clinical opinion. Further, the instruments were designed to be suitable for a variety of indications where an acute medical event could occur and in diverse research settings (e.g., real-world evidence studies, single-arm clinical trials, randomized controlled trials).

## Methods

### Study design

This was an instrument development and content validation study that involved literature review, clinical expert advice, and concept elicitation (CE) and cognitive debriefing (CD) interviews with English-speaking patients and clinicians based in the United States of America (USA), United Kingdom (UK), and Canada. Two instruments, the Patient Satisfaction with Diagnostic Test Questionnaire (PSDT-Q) and the Clinician Satisfaction with Diagnostic Test Questionnaire (CSDT-Q), were developed based on the findings from literature review, CE interviews, and input from clinical experts, then evaluated for content validity in CD interviews.

**Fig. 1:**
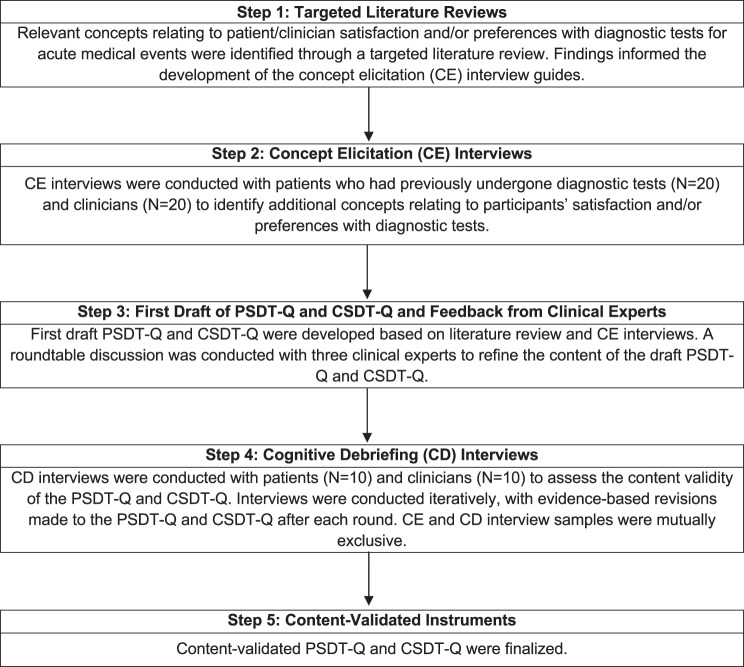
Summary of PSDT-Q and CSDT-Q development and content validation

The development and content validation followed best practice guidance for instrument development [29–32]. Fig. 1 provides a summary of the development and content validation of the instruments.

### Study participants

Patients and clinicians based in the USA, UK, and Canada were interviewed for the CE and CD interview steps of instrument development. Patients were eligible for the study if they were aged 18+ years and had previously undergone a diagnostic evaluation for an acute medical event, including a respiratory infection, sexually transmitted genital tract infection (STI), cardiovascular disease (CVD) related event, or acute diarrhea, in the three months prior to interview screening. These conditions were selected to reflect a variety of diagnostic methods and a range of indications in which an acute medical event occurs. Eligible clinicians were required to have at least two years of experience performing diagnostic evaluations in one of these disease indications, and to administer at least five diagnostic tests per month within this indication. This refers to having had involvement in the diagnostic test process (i.e., ordering diagnostic tests; or collecting specimen to send for diagnostic results; or performing tests and determining the diagnostic results themselves) and using the results in clinical decision-making. For both the patient and clinician interviews, the CE and CD samples were mutually exclusive. Demographic data (e.g., race, ethnicity, sex, gender) were collected to understand the composition of the samples recruited. Further, recruitment targets for disease indications and demographic characteristics were used to ensure findings captured diverse experiences. The full inclusion and exclusion criteria are outlined in Supplementary Material A.

All participants were recruited through a specialist recruitment agency utilizing various sources, including patient advocacy groups and participant databases. Participant eligibility was confirmed using a screening questionnaire. All participants provided informed consent to participate in an interview. All interviews were conducted by videoconference (Zoom) in English by experienced qualitative researchers (KW, EE, GT and CD). Interviews lasted approximately 60 minutes, were audio-recorded, transcribed verbatim, and de-identified prior to analysis. Participants were remunerated for their time via the specialist recruitment agency in line with fair market value in the country from which they were recruited.

The study was reviewed by the Western Institutional Review Board-Copernicus Group (WCG) institutional review board and received an exempt status determination on March 30th, 2022.

### Development and content validation process

#### Targeted literature review and concept elicitation interviews

Targeted literature reviews were conducted to inform the development of the CE interview materials (Supplementary Material B). Separate semi-structured interview guides were developed for the patient and clinician interviews. The interview guides included questions to explore factors that impacted the experience and satisfaction with the diagnostic evaluation(s) that participants had either engaged in or administered.

Demographic data from the background questionnaire and screener completed by the CE interview participants were summarized using descriptive statistics. Data from the patient and clinician interviews were analyzed using thematic and content analysis [33, 34] in the MAXQDA software package [35]. Separate coding frameworks were developed for the patient and clinician analyses based on the concepts included in the interview guide and reported in the interviews. Data were coded by key concepts (i.e., concepts relating to factors that impact clinicians’ and patients’ satisfaction and preferences towards diagnostic testing), with further subcodes applied depending on if the concept was first reported by the participant spontaneously (S), when probed (P), if the evidence was not clear (NC), if no impact on satisfaction was reported (N) or if the concept was not discussed in the interview (NA). Two researchers, both of whom conducted the interviews, independently coded a selection of interview transcripts. Coding was compared and, once an agreement on the initial coding framework was reached, subsequent interviews were then coded. An iterative coding approach was followed, moving between consecutive transcripts and new codes that emerged. A quality review of the coding framework and coded data was conducted by a senior research team member.

To ensure the sample size was sufficient and all relevant themes were captured, conceptual saturation was monitored using a saturation grid. Conceptual saturation was defined as the point at which no new concepts were elicited in the interviews (i.e., all reported concepts had been discussed in previous interviews) [36]. Identified concepts were used to inform the development of the draft PSDT-Q and CSDT-Q.

#### Input from clinical experts

Three clinical experts (MM, RB and LK) with extensive experience with diagnostics in the target indications provided their feedback on the draft PSDT-Q and CSDT-Q in two roundtable meetings hosted via videoconference (Zoom). Input from clinical experts was used to determine the suitability and relevance of draft content, and to revise as necessary ahead of assessment in CD interviews.

#### Cognitive debriefing (CD) interviews

The content validity of the PSDT-Q and CSDT-Q were evaluated in CD interviews. Structured cognitive debriefings of the PSDT-Q (patients) or CSDT-Q (clinicians) explored participants’ understanding of each item, the relevance of the item to their satisfaction with diagnostic tests in the indication of interest, appropriateness of the response options, and conceptual comprehensiveness of the instruments.

Demographic data collected from the screeners completed by CD interview participants (containing the same questions as the CE interview screener and background questionnaire) were summarized using descriptive statistics. Patient and clinician interview data were extracted into analysis frameworks in Microsoft Excel. Within these analysis frameworks, data relating to instruction/item understanding, concept relevance, appropriateness of response options, and conceptual comprehensiveness were coded. General feedback was summarized narratively. The populated extraction frameworks were continuously examined to determine the need to revise the PSDT-Q or CSDT-Q content. A quality review of the populated analysis framework was conducted by a senior researcher.

CD interviews were conducted in two iterative rounds (*N* = 5 per round, per group). Evidence-based revisions were made to the instruments after each round. Minor revisions were made to the PSDT-Q part way through round 2 (after *n* = 2/5 interviews).

## Results

### Study participant characteristics

CE interviews were conducted with patients and clinicians (*N* = 20 each) between April and July 2022. CD interviews were conducted with patients and clinicians (*N* = 10 each) between January and July 2023. Represented indications in the patient and clinician samples included respiratory infections, STIs, CVDs, and acute diarrhea, countries included Canada, UK, and USA, and diagnostic evaluations included sample-based tests (blood, urine, throat or nasal swab, genital tract swab, phlegm sample, stool sample), imaging, physical examination, and clinician diagnosis based on symptoms. Patient and clinician characteristics are presented in Tables 1 and 2, respectively.

**Table 1:** Patient sample characteristics for the CE* and CD^†^ interviews

Characteristic	CE interviews (N = 20)	CD interviews (N = 10)
Age - Mean (Min, Max)	42.9 (27, 60)	44 (20, 72)
Sex - N (%)		
Female	16 (80)	7 (70)
Male	4 (20)	2 (20)
Prefer to self-describe	0 (0)	1 (10)
Country of residence - N (%)		
Canada	9 (45)	3 (30)
USA	8 (40)	4 (40)
UK	3 (15)	3 (30)
Race - N (%)		
White	7 (35)	8 (80)
Black	4 (20)	0 (0)
Asian	2 (10)	1 (10)
African American	2 (10)	0 (0)
American Indian/Alaskan Native	0 (0)	1 (10)
Hispanic/Latino	2 (10)	0 (0)
Indigenous	1 (5)	0 (0)
South Asian	1 (5)	0 (0)
Mixed race of multiple ethnic groups	1 (5)	0 (0)
Diagnostic area discussed in interview^‡^ - N (%)		
Influenza	3 (15)	2 (20)
COVID-19	3 (15)	3 (30)
Gonorrhea	2 (10)	1 (10)
Chlamydia	2 (10)	2 (20)
Other sexually transmitted genital tract infection	3 (15)	1 (10)
Heart failure	4 (20)	3 (30)
Myocardial infarction	2 (10)	0 (0)
Acute diarrhea	1 (5)	1 (10)
Diagnostic evaluations discussed in interview^‡^ - N (%)		
Blood test	8 (40)	NA
X-ray or CT scan	7 (35)	NA
Urine sample	6 (30)	NA
Throat or nasal swab	4 (20)	NA
Physical examination	4 (20)	NA
Genital tract swab	4 (20)	NA
Ultrasound	4 (20)	NA
Echocardiogram	3 (15)	NA
Clinician diagnosis based on symptoms	2 (10)	NA
Electrocardiogram	2 (10)	NA
MRI	1 (5)	NA
Angiography	1 (5)	NA
Stool sample	1 (5)	NA
Phlegm sample	1 (5)	NA

^*^ CE interviews: Concept elicitation (CE) interviews were conducted with patients who had previously undergone diagnostic tests (*N* = 20) to identify additional concepts relating to participants’ satisfaction and/or preferences with diagnostic tests

^†^ CD interviews: Cognitive debriefing (CD) interviews were conducted with patients (*N* = 10) to assess the content validity of the PSDT-Q. Interviews were conducted iteratively, with evidence-based revisions made to the PSDT-Q after each round. CE and CD interview samples were mutually exclusive

‡ The sum of percentages does not equal 100 as some participants discussed their experience with more than one diagnostic evaluation during the interview

Abbreviations: COVID-19 = coronavirus disease 2019; CT = computed tomography; MRI = magnetic resonance imaging; *N* = Number of participants; NA = not applicable; UK = United Kingdom; USA = United States of America

**Table 2:** Clinician sample characteristics for the CE^*^ and CD^†^ interviews

Characteristic	CE interviews (N = 20)	CD interviews (N = 10)
Experience performing tests (years) - Mean (Min, Max)	10.1 (2, 30)	24.6 (7, 37)
Tests performed per month - Mean (Min, Max)	103.3 (10, 500)	134.7 (12, 800)
Country - N (%)		
UK	7 (35)	3 (30)
USA	7 (35)	4 (40)
Canada	6 (30)	3 (30)
Job title - N (%)		
General practitioner [or primary care practitioner]	10 (50)	5 (50)
Internal medicine physician or internist	4 (20)	2 (20)
Urgent care doctor or emergency doctor	4 (20)	2 (20)
Nurse practitioner [or nurse]	2 (10)	1 (10)
Used POC tests - N (%)		
Yes	17 (85)	8 (80)
No	3 (15)	2 (20)
Diagnostic indication discussed in interviews^‡^ - N (%)		
COVID-19	7 (35)	1 (10)
Influenza	3 (15)	0 (0)
Other upper and lower respiratory tract infections	0 (0)	1 (10)
Chlamydia	6 (30)	3 (30)
Gonorrhea	5 (25)	0 (0)
Mycoplasma genitalium	3 (15)	0 (0)
Heart failure	5 (25)	2 (20)
Myocardial infarction	4 (20)	2 (20)
Acute diarrhea	3 (15)	1 (10)

^*^ CE interviews: Concept elicitation (CE) interviews were conducted with clinicians (*N* = 20) to identify additional concepts relating to participants’ satisfaction and/or preferences with diagnostic tests

^†^ CD interviews: Cognitive debriefing (CD) interviews were conducted with clinicians (*N* = 10) to assess the content validity of the CSDT-Q. Interviews were conducted iteratively, with evidence-based revisions made to the CSDT-Q after each round. CE and CD interview samples were mutually exclusive

^‡^ The sum of percentages does not equal 100 as some clinicians discussed their experience testing for more than one diagnostic indication during the interview

Experience performing tests (years) = Number of years of experience performing diagnostic tests in the primary diagnostic area

Tests per month = Average number of diagnostic tests in primary diagnostic area per month

Text in brackets indicates criteria updated for use in CD interviews only

Abbreviations: COVID-19 = coronavirus disease 2019; *N* = number of participants; POC = point-of-care; UK = United Kingdom; USA = United States of America

### CE results

During the CE interviews, patients and clinicians reported a wide range of concepts impacting their satisfaction with diagnostic tests. Of note, all patients reported that results wait time, geographical location where the test took place, and convenience impacted their satisfaction with diagnostic tests, while all clinicians reported ease of results interpretation and ease-of-use as impacting factors. An overview of concepts reported by patients and clinicians, as well as illustrative quotes, are provided in Tables 3 and 4, respectively. Concept saturation was met in the patient and clinician samples (Saturation Grids, Supplementary Material C).

**Table 3:** Demonstrative quotes: patient-reported concepts impacting satisfaction with diagnostic tests (*N* = 20)

Concept	Total n (%)	Illustrative quote
Result wait time	20 (100)	*“I was quite satisfied because the results, they said it would take 48 hours and it didn’t, it came sooner …”* – P1-UK-RESP-CE, discussing a throat/nasal swab (PCR)
Geographical location	20 (100)	*“I was very satisfied because it wasn’t far and I was familiar already with the location”* – P3-USA-RESP-CE, discussing a throat/nasal swab (PCR)
Convenience	20 (100)	*“… you carry the bottle, put it in a sanitized sort of bag and then from there carry it to the lab where you have to drop it off. So it was convenient and easy”* – P15-CAN-AD-CE, discussing a stool sample
Administration time	19 (95)	“*I wish it had been quicker, but I mean it takes that length of time to check the arteries, the veins, the heart, the muscle.*”– P16-USA-CVD-CE, discussing a CT scan
Accuracy	18 (90)	*“I feel very confident that the results were accurate, because I’m dealing with a major hospital and so they’re pretty renowned medical professionals …”* – P18-CAN-STI-CE, discussing a urine sample and genital swab
Information provision	18 (90)	“*I was satisfied with it and the doctor or physician was very informative, he told me what he was testing me for, he told me the timeframe on my results, and he told me the purpose of, so it was very informative, very transparent. I wasn’t wondering, “Why are we doing this?” He answered all my questions …*” – P12-CAN-STI-CE, discussing a urine sample
Method of result delivery	17 (85)	*“Very satisfied, so my doctor would usually just go over the results with me and like if I had any questions or anything I didn’t understand he would go over them thoroughly.”* – P10-CAN-RESP-CE, discussing a blood test and clinician diagnosis based on symptoms
Hygiene	16 (80)	*“… the blood test and the urine test were done … the doctor … called the nurse in … she cleaned my arm, and it was all hygienic and everything. So yeah, everything was newly used.”* – P4-UK-RESP-CE, discussing a blood test and urine sample
Sample collection method	15 (75)	*“I don’t like needles, so that was very good. It wasn’t invasive. Physically it was non-invasive.”* – P20-CAN-CVD-CE, discussing an electrocardiogram
Impact on emotional well-being: result wait time	9 (45)	*“I would say like while waiting on the results, like I was kind of worried, like would it be a positive test or a negative test?” –* P7-USA-RESP-CE, discussing a throat/nasal swab
Impact on clinical pathway	9 (45)	*“We didn’t know I had an enlarged heart. So that gave them the knowledge to say, hey look, we’ve got to elevate this, and we’ve got to take this away. We’ve got to add this, ACE inhibitors and stuff. They had to add some stuff to my medicine”* – P16-USA-CVD-CE, discussing a CT scan
Ease-of-use	8 (40)	*“I think the CT evaluation was the most convenient of all. You literally just go in there and lay down, either get the IV or don’t get the IV, don’t move, and you’re done. It’s [CT scan] the most easiest diagnostic test there is”* – P9-USA-CVD-CE, discussing a CT scan
Appearance	6 (30)	*“Yes, how everything is separated in bags … it’s very clean, organized and separated by bags”* – P2-CAN-RESP-CE, discussing a throat/nasal swab (Antigen test)
Impact on emotional well-being: sample collection method	5 (25)	*“It causes me anxiety and I hyperventilate sometimes … But it’s just the thought of that thing not opening. I know that sounds crazy, but that thing not opening and me suffocating”* – P16-USA-CVD-CE, discussing a CT scan

n = number of patient CE interview respondents who indicated that the concept impacted their satisfaction

**Abbreviations**: ACE inhibitor = angiotensin-converting enzyme inhibitor; CAN = Canada; CT = computed tomography; CE = concept elicitation; COVID-19 = coronavirus disease 2019; CVD = cardiovascular disease; RESP = respiratory infections, STI = sexually transmitted infection; UK = United Kingdom; USA = United States of America; PCR = polymerase chain reaction

**Table 4:** Demonstrative quotes: clinician-reported concepts impacting satisfaction with diagnostic tests (*N* = 20)

Concept	Total n (%)	Illustrative quote
Ease of results interpretation	20 (100)	*“If it’s done properly yes, very satisfied. The machine gives a good printout, and also it interprets the electrocardiogram automatically.”* – IM11-USA-CVD/STI-CE, discussing an electrocardiogram
Ease-of-use	20 (100)	*“I mean definitely the urine is very, very simple. So that’s very satisfying. As I say, the swab testing is more arduous”* – GP10-UK-RESP/STI-CE, discussing a urine sample and genital swab
Result wait time	19 (95)	*“Generally, you get the results within I think even for MRIs, you get them within seven days I think, within a week, so it’s not really waiting too long”* – GP14-CAN-CVD-CE, discussing an MRI
Impact on patients’ clinical pathway	19 (95)	“*… it can either allow for us to be on the right route or it can allow for us to change treatment if we haven’t put them on …*” – GP19-CAN-AD/RESP-CE, discussing a stool sample
Convenience	18 (90)	*“… In one area that I work in I can request an ECG with a couple of clicks of my mouse.”* – GP9-UK-CVD-CE, discussing an electrocardiogram
Accuracy	17 (85)	*“… we found some people with symptoms were coming up negative and we were sure they had COVID, and we were finding people without symptoms were coming up positive who then turned out on PCR not to have COVID.”* – UC6-UK-RESP-CE, discussing a throat/nasal swab (Antigen/PCR)
Guidance and training	16 (80)	“*… I think there could be more training available to patients and healthcare providers on the performance of the testing …*” – NP12-CAN-RESP-CE, discussing a nasal swab (Antigen/PCR)
Administration time	14 (70)	*“Very rapid. I mean we have assistants, we have MAs [medical assistants]. They’re able with the cooperative patients to do them in minutes or less.”* – UC3-USA-STI-CE, discussing a urine sample and genital swab
Financial cost	13 (65)	*“Cost is certainly an issue.”* – GP15-CAN-RESP/CVD-CE, discussing PCR and antigen tests

n = number of clinician CE interview respondents who indicated that the concept impacted their satisfaction

Abbreviations: AD = acute diarrhea; CAN = Canada; CE = concept elicitation; CVD = cardiovascular disease; ECG = electrocardiogram; GP = general practitioner; IM = internal medicine physician or internist; NP = nurse practitioner; RESP = respiratory infections; STI = sexually transmitted infection; UC = urgent care or emergency doctor, UK = United Kingdom; USA = United States of America; PCR = polymerase chain reaction

### Input from clinical experts

Three clinical experts (MM, RB, and LK) with extensive experience in diagnostics provided feedback on the draft PSDT-Q and CSDT-Q to determine the suitability and relevance of draft content. Revisions were implemented based on the feedback (Supplementary Material D).

### CD results

#### PSDT-Q

An overview of the PSDT-Q following CD interviews can be found in Table 5.

**Table 5:** Overview of the PSDT-Q

PSDT-Q item	Concept	Response scale
**Section 1** **– Satisfaction with diagnostic test process**		
1.1	Information provision	5-point Likert, Satisfaction scale
1.2	Ease of completion	
1.3	Time taken to perform	
1.4	Speed of results	
1.5	Method of result delivery	5-point Likert, Satisfaction scale, ‘I did not receive my test result’
1.6	Ease of understanding	
1.7	Accuracy	
1.8	Impact on clinical pathway	5-point Likert, Satisfaction scale, N/A
1.9	Overall satisfaction	5-point Likert, Satisfaction scale
**Section 2** – **Satisfaction with sample or measurement collection**		
2.1	Comfort	5-point Likert, Satisfaction scale
2.2	Convenience	
2.3	Type of sample or measurement	
2.4	Person collecting sample or measurement	
2.5	Hygiene	
**Section 3** – **Emotional well-being during diagnostic test process**		
3.1	Overall	0–10 Numeric response scale with illustrative verbal anchors, Emotional well-being scale
3.2	Sample or measurement collection	0–10 Numeric response scale with illustrative verbal anchors, Emotional well-being scale, N/A
3.3	Result wait time	0–10 Numeric response scale with illustrative verbal anchors, Emotional well-being scale
**Section 4** – **Preferences for diagnostic tests [Optional add-on module]**		
4.1	Overall preference	5-point Likert, Preference scale
4.2	Future willingness	5-point Likert, Comparable willingness scale
4.3	Comparable confidence	5-point Likert, Comparable confidence scale

N/A = Not applicable response option

The PSDT-Q instructions and sections 1–3 headers were understood by most patients across rounds leading to no revisions. The section 4 header was revised following round 2 to clarify that items in this section were designed to compare two tests.

Evidence-based revisions were made to specific items and response scales based on respondent feedback. Following round 1, eight items (1.2, 1.3, 1.5, 1.8, 2.2, 2.3, 2.4, 2.5; Table 5) and four response scales (Satisfaction scale with ‘I did not receive my test result’ (1.5–1.7); Preference scale (4.1); Comparable willingness scale (4.2); Comparable confidence scale (4.3)) were revised. Three items (3.1, 3.2, 3.3) assessing the emotional well-being during a diagnostic test on a numeric response scale (NRS) were added following round 1 and two items assessing emotional well-being on a Likert scale were subsequently removed following round 2, as the equivalent NRS items were better understood by patients.

Interim revisions were performed during round 2: a minor wording modification was made to item 3.1, and two new items (2.1, 3.2) were added. These interim revisions were debriefed by the final three round 2 patients (*n* = 3/5). Following round 2, minor wording revisions were made to item 2.1, and one response scale (Satisfaction scale, not applicable [N/A] (1.8)) was amended to improve clarity. Key words in item 3.1 were bolded for emphasis. Two items (1.2, 1.8) were reverted to their original wording due to a preferable performance in round 1.

Eight items (1.1, 1.4, 1.6, 1.7, 1.9, 4.1, 4.2, 4.3) and one response scale (Satisfaction scale (1.1–1.5, 1.9, 2.1–2.5)) required no revisions across rounds. Examples of the PSDT-Q iterative revision process are included in Supplementary Material E1. Further, an item tracking matrix summarizing all evidence-based revisions made to the PSDT-Q across rounds is provided in Supplementary Material F1. Overall, the concepts included in the PSDT-Q were found to be of acceptable relevance to patients and evidence-based revisions improved patient understanding.

#### CSDT-Q

An overview of the CSDT-Q following CD interviews can be found in Table 6.

**Table 6:** Overview of the CSDT-Q

CSDT-Q item	Concept	Response scale
**Section 1**– **Satisfaction with the ‘test’ and ‘standard care’**		
1	Ease-of-use	5-point Likert, Satisfaction scale, N/A
2	Time taken to perform	
3	Speed of results	
4	Ease of results interpretation	
5	Impact on care plan	
6	Impact on consultations	
7	Impact on workload/workflow	
8	Guidance and training	
**Section 2** – **Confidence in the ‘test’ and ‘standard care’**		
9	Accuracy	5-point Likert, Confidence scale, N/A
**Section 3** – **Preference for the ‘test’ versus ‘standard care’**		
10	Overall preference	5-point Likert, Preference scale, N/A
11	Future willingness	5-point Likert, Comparable willingness scale, N/A
12	Comparable confidence	5-point Likert, Comparable confidence scale, N/A

N/A = Not applicable response option

No revisions were made to the CSDT-Q instructions and sections 2 and 3 headers as they were understood by most clinicians across rounds. Following round 2, the section 1 header was revised to clarify when the ‘not applicable’ response option should be selected.

No revisions were made to the CSDT-Q following round 1 as content was understood and conceptually relevant to most clinicians. Round 1 feedback was used to inform revisions to the interview guide made prior to round 2. Following round 2, minor wording revisions were made to two items (1, 8; Table 6) to improve clarity. No revisions were required to the remaining ten items or any response scales. Examples of the CSDT-Q iterative revision process are included in Supplementary Material E2 and an item tracking matrix summarizing all evidence-based revisions made to the CSDT-Q across rounds is provided in Supplementary Material F2.

#### Content-validated PSDT-Q and CSDT-Q

The content-validated PSDT-Q consisted of four sections (20 items total) and assessed satisfaction with the diagnostic test process (section 1), satisfaction with sample or measurement collection (section 2), emotional well-being during the diagnostic test process (section 3), and preference between two diagnostic tests (section 4, optional add-on module) [Table 5]. The content-validated CSDT-Q consisted of three sections (12 items total) and assessed satisfaction (section 1), confidence (section 2), and preferences (section 3) for the diagnostic test under study versus standard care [Table 6].

## Discussion

This study resulted in the development of two novel clinical outcomes assessment (COA) instruments to measure satisfaction with diagnostic tests among patients and clinicians. These instruments, the Patient Satisfaction with Diagnostic Test Questionnaire (PSDT-Q) and the Clinician Satisfaction with Diagnostic Test Questionnaire (CSDT-Q), are the first validated tools designed specifically to assess satisfaction with diagnostic tests for acute medical events across various disease areas and research environments.

Explicit consideration of patient experience and acceptability has often been neglected in diagnostic evaluations, with evidence generation focusing mainly on accuracy, overlooking other test attributes such as patient and clinician satisfaction [37]. When satisfaction is explored, often the experience of diagnostic services can be conflated with the experience and acceptability of diagnostic tests [38]. Here, we provide a tool to explicitly explore patient satisfaction with the test, separating this from the diagnostic service. Both the PSDT-Q and CSDT-Q incorporate a holistic evaluation of the testing process, accounting for the factors that were identified as being most important to patients and clinicians including, for example, convenience of testing and the impact the result will have on the patient’s ongoing care.

By measuring patient and clinician satisfaction with diagnostic tests, the PSDT-Q and CSDT-Q instruments can provide valuable insights into the lived experience of individuals in these populations. These instruments provide means for researchers to improve the efficiency of their research, ensuring optimal data capture. Such data may prove instrumental in HTA submissions for diagnostic tests to support reimbursement decisions or funding recommendations. For example, this data can shed light on patient preferences, values, or needs related to health conditions or interventions [27]. These instruments can capture insights from patients and clinicians that cannot be quantified through health economic modeling alone. Moreover, existing observational evidence in the literature strongly suggests that patient and clinician satisfaction influences the successful adoption of medical tests [10, 13, 14, 16, 17, 39]. This underscores the importance of the PSDT-Q and CSDT-Q in providing data on individuals’ lived experiences, which may be crucial for assessing the true value of novel diagnostics.

Patient and clinician satisfaction may also have important economic implications. For example, greater satisfaction with diagnostic tests may lead to better adherence to testing protocols and overall care, resulting in treatment that is more appropriate towards improved clinical outcomes [40, 41]. Therefore, capturing satisfaction data using instruments like the PSDT-Q and CSDT-Q is crucial. Future research should examine the association between these instruments and outcomes and costs, while advocating for a modern HTA framework that better considers satisfaction metrics like the ones proposed in this study. This approach could enhance the evaluation of diagnostic tests, ultimately improving patient care and economic efficiency.

This study had limitations that should be acknowledged. First, as only patients and clinicians who underwent diagnostic evaluations or conducted diagnostic evaluations for acute medical events (respiratory infections, STIs, CVDs, and acute diarrhea) were included in the current study, additional research may be required to determine the content validity of these instruments in chronic conditions. Second, sample sizes in the CD interviews were relatively small (10 patients and 10 clinicians), which may limit the generalizability of the study findings. Third, the questionnaires were developed through interviews with patients and clinicians who speak English so some elements of satisfaction may differ depending on cultural differences, which have not been explored through this work. This could lead to a limitation of the applicability of these questionnaires in future research. Fourth, while the PSDT-Q and CSDT-Q tools are designed to apply to all diagnostic tests but with particular emphasis on settings where acute medical events may occur, the questionnaires may require some customization for application in other specific settings. This approach is common when applying other tools, such as when assessing the risk of bias in systematic reviews.

The psychometric validity of the PSDT-Q and CSDT-Q has yet to be established. Ongoing research seeks to investigate the psychometric properties of the instruments, including exploring potential scoring algorithms and the reliability and validity in select indications. The PSDT-Q and CSDT-Q may be further refined (e.g., item reduction) based on the findings of this psychometric research.

In the future, the PSDT-Q and CSDT-Q could be used in conjunction with evidence frameworks, such as those presented by Horvath et al. 2014 [42] and Ferrante di Ruffano et al. 2012 [43], which explicitly acknowledge that patient and clinician experience can affect both downstream health behaviors and clinical behavior, respectively. The PSDT-Q instrument could additionally support gathering data on patient acceptability for structured evaluation tool kits such as Point-of-Care Key Evidence Tool (POCKET): a checklist for multi-dimensional evidence generation in POC tests [44].

While these tools have been initially designed for diagnostic tests, with an eye on acute conditions, many of the concepts are likely to be relevant to other diagnostic modalities such as digital health and chronic conditions where place-based care, such as at home and community testing, may be relevant, particularly in the context of satisfaction. Future research should evaluate the suitability of the PSDT-Q and CSDT-Q for use in these settings and explore potential adaptation as needed to suit these contexts. These instruments will also benefit from further use in different study types, such as observational versus interventional studies. Additionally, further work should look to translate and validate the instruments in different languages to support health equity considerations.

We encourage evaluators to leverage the PSDT-Q and CSDT-Q to incorporate patient and clinician perspectives in the evaluation process for diagnostic tests. Integrating these instruments into assessment frameworks can ensure that the preferences and values of those directly impacted by diagnostic innovations are represented. This approach can enhance the comprehensiveness and relevance of evaluations, resulting in more informed healthcare decisions. We encourage stakeholders to embrace this opportunity, incorporating thorough consideration of patient and clinician perspectives to shape the future of clinical diagnostics. By doing so, we can drive advancements that truly reflect the needs and values of the healthcare community.

## Conclusions

The PSDT-Q and CSDT-Q are the first content-validated instruments designed to evaluate patient and clinician satisfaction and preferences for diagnostic tests. Ongoing research is exploring the psychometric properties of the instruments, scoring algorithms, and use in different indications. Alongside clinical and economic data, data derived from the PSDT-Q and CSDT-Q can offer valuable supplementary information from the perspective of patients and clinicians, bridging gaps and providing context for decision-makers when evaluating novel diagnostic tests. Further integrating evidence-based measurement tools, such as the PSDT-Q and CSDT-Q, into clinical studies, value assessment frameworks, and health technology evaluations can inform the evolution of diagnostic tests and can ensure that the preferences and values of those directly impacted by diagnostic innovations are represented. This process can facilitate the incorporation of diverse viewpoints into healthcare decision-making, advancing the trajectory towards more individualized and experience-centered healthcare.

## Electronic supplementary material

Below is the link to the electronic supplementary material.

Supplementary Material 1

## Data Availability

The datasets used and/or analyzed during the current study are available from the corresponding author on reasonable request.

## List of abbreviations

ACE inhibitor: Angiotensin-converting enzyme inhibitor
AD: Acute diarrhea
CAN: Canada
CD: Cognitive debriefing
CE: Concept elicitation
COA: Clinical outcome assessment
COVID-19: Coronavirus disease 2019
CSDT-Q: Clinician Satisfaction with Diagnostic Test Questionnaire
CT scans: Computed tomography scans
CVD: Cardiovascular disease
ECG: Electrocardiogram
HPV: Human papillomavirus
HTA: Health technology assessment
MRI: Magnetic resonance imaging
N/A: Not applicable
NRS: Numeric response scale
PCR: Polymerase chain reaction
POC: Point-of-care
POCKET: Point-of-Care Key Evidence Tool
PRO: Patient-reported outcome
PSDT-Q: Patient Satisfaction with Diagnostic Test Questionnaire
RESP: Respiratory infections
STI: Sexually transmitted infection
UK: United Kingdom
USA: United States of America
WCG: Western Institutional Review Board-Copernicus Group
