# Supplementary material for: Beyond diagnostic test performance: two content-validated questionnaires assessing patient and clinician satisfaction with diagnostic tests
Source: J Patient Rep Outcomes. 2025 Nov 27;10:9. doi: 10.1186/s41687-025-00964-4 (PMC12816492; doi:10.1186/s41687-025-00964-4)
Supplement: Supplementary file 1 — Supplementary Material 1 [file 41687_2025_964_MOESM1_ESM.docx]

Supplementary Material

Table of Contents

[Supplementary Material A – Participant concept elicitation (CE) and cognitive debriefing (CD) inclusion criteria 2](#_Toc199930288)

[Supplementary Material A1: Patient inclusion criteria for concept elicitation (CE) and cognitive debriefing (CD) interviews **2**](#_Toc199930289)

[Supplementary Material A2: Clinician inclusion criteria for concept elicitation (CE) and cognitive debriefing (CD) interviews **3**](#_Toc199930290)

[Supplementary Material B – Targeted Literature Review 4](#_Toc199930291)

[Supplementary Material B1: Search strings **5**](#_Toc199930292)

[Supplementary Material B2: Inclusion / exclusion criteria (Search 1 and 2) **7**](#_Toc199930293)

[Supplementary Material B3: Flow diagram of literature screening **8**](#_Toc199930294)

[Supplementary Material B4: Targeted literature review findings **9**](#_Toc199930295)

[Supplementary Material C – Data saturation: patient and clinician concept elicitation (CE) interviews 11](#_Toc199930296)

[Supplementary material C1: Saturation matrix for patient concept elicitation (CE) interviews **11**](#_Toc199930297)

[Supplementary Material C2: Saturation matrix for clinician concept elicitation (CE) interviews………………………………………………………………………………………………………. **12**](#_Toc199930298)

[Supplementary Material D – Summary of clinical expert feedback on the first draft Patient Satisfaction with Diagnostic Test Questionnaire (PSDT-Q) and Clinician Satisfaction with Diagnostic Test Questionnaire (CSDT-Q) 13](#_Toc199930299)

[Supplementary Material D1: Summary of clinical expert feedback on the first draft Patient Satisfaction with Diagnostic Test Questionnaire (PSDT-Q) **13**](#_Toc199930300)

[Supplementary Material D2: Summary of clinical expert feedback on the first draft Clinician Satisfaction with Diagnostic Test Questionnaire (CSDT-Q) **14**](#_Toc199930301)

[Supplementary Material E – Example revisions to the Patient Satisfaction with Diagnostic Test Questionnaire (PSDT-Q) and Clinician Satisfaction with Diagnostic Test Questionnaire (CSDT-Q) based on interview findings 15](#_Toc199930302)

[Supplementary Material E1: Example revisions to the Patient Satisfaction with Diagnostic Test Questionnaire (PSDT-Q) based on patient cognitive debriefing interviews **15**](#_Toc199930303)

[Supplementary Material E2: Example revisions to the Clinician Satisfaction with Diagnostic Test Questionnaire (CSDT-Q) based on clinician cognitive debriefing interviews **17**](#_Toc199930304)

[Supplementary Material F – Patient Satisfaction with Diagnostic Test Questionnaire (PSDT-Q) and Clinician Satisfaction with Diagnostic Test Questionnaire (CSDT-Q) item tracking matrices 19](#_Toc199930305)

[Supplementary Material F1: Patient Satisfaction with Diagnostic Test Questionnaire (PSDT-Q) item tracking matrix: Summary of participant feedback that informed evidence-based revisions **19**](#_Toc199930306)

[Supplementary Material F2: Clinician Satisfaction with Diagnostic Test Questionnaire (CSDT-Q) item tracking matrix: Summary of participant feedback that informed evidence-based revisions **22**](#_Toc199930307)

# Supplementary Material A – Participant concept elicitation (CE) and cognitive debriefing (CD) inclusion criteria

## Supplementary Material A1: Patient inclusion criteria for concept elicitation (CE) and cognitive debriefing (CD) interviews

| Patient Inclusion Criteria (CE & CD interviews) |
| --- |
| - Aged 18+ years - Live in the UK, USA, or Canada - Sufficient English language proficiency to participate in an interview - Undergone a diagnostic evaluation in the past three months at the time of screening for one of the following conditions: COVID-19, influenza, a sexually transmitted genital tract infection (including chlamydia, gonorrhea, mycoplasma genitalium and / or another sexually transmitted genital tract infection), acute diarrhea, acute deterioration of chronic heart failure (e.g., edema) or myocardial infarction - Willing and able to give their informed consent to take part in a 60-minute audio-recorded interview - CD interview only: Have not previously participated in a CE interview for this study   Abbreviations: COVID-19 = coronavirus disease 2019; UK = United Kingdom; USA = United States of America |

## Supplementary Material A2: Clinician inclusion criteria for concept elicitation (CE) and cognitive debriefing (CD) interviews

| Clinician Inclusion Criteria (CE & CD interviews) |
| --- |
| - Live in the UK, USA, or Canada - Currently a licensed general practitioner, emergency medicine doctor or urgent care doctor, internal medicine doctor, or nurse practitioner - Experience using diagnostic tests in clinical decision-making for acute medical events in one of the following disease areas: cardiovascular diseases (including heart failure and / or myocardial infarction), STIs (including chlamydia, gonorrhea and / or mycoplasma genitalium), acute diarrhea or respiratory infections (including influenza or COVID-19) - At least two years of experience performing diagnostic tests for at least one of the target indications - Administers at least five diagnostic tests per month for at least one of the target indications - Willing and able to take part in a 60-minute audio recorded interview - CD interview only: Have not previously participated in a CE interview for this study   Abbreviations: COVID-19 = coronavirus disease 2019; UK = United Kingdom; USA = United States of America |

# Supplementary Material B – Targeted Literature Review

Targeted literature reviews focused on identifying key concepts relating to patient and clinician satisfaction with diagnostic evaluations (including point of care [POC] tests). Two searches were conducted on Ovid in MEDLINE, Embase and PsychInfo in January 2022. Supplementary searches were also conducted in Google Scholar.

Search 1 aimed to identify publications / reviews describing patient and / or clinician satisfaction and preferences with POC tests. Search 2 aimed to identify publications describing the development and / or validation of a patient-reported outcome (PRO) or clinician-reported instrument.

## Supplementary Material B1: Search strings

Table 1. Ovid search terms - Search 1: Satisfaction and preference with POC tests

| No. | Search Terms |
| --- | --- |
| 1 | COVID* or SARS-CoV-2 or Influenza or flu or myocardial infarction or heart failure or STI or STD or sexually transmitted* or infectious disease* or cardiovascular* or acute exacerbation or mycoplasma genitalium or chlamydia or gonorrhea or gonorrhoea or Group A strep* or pharyngitis or strep A or respiratory [abstract or title] |
| 2 | acute* or emergency [abstract or title] or (point of care* or point-of-care* or POCT or rapid test*) or (point of care and medical device*) or (point of care and diagnostic test*) or (point-of-care and medical device*) or point-of-care and diagnostic test* [abstract or title] |
| 3 | satisfaction or preference* or perception* or attitude* or experience* or interview* or qualitative or usability [abstract or title] |
| 4 | **1 AND 2 AND 3** |

Abbreviations: POC = point-of-care; POCT = point-of-care tests; COVID = coronavirus; STI = sexually transmitted infection; STD = sexually transmitted disease

Table 2. Google scholar terms – Search 1: Reviews of satisfaction / preference with diagnostic testing

| No. | Search Terms |
| --- | --- |
| 1 | “point of care” “satisfaction” ”review” |
| 2 | “point of care” “preference” ”review” |

Table 3. Ovid search terms - Search 2: PRO / clinician-outcome instrument development / validation

| No. | Search Terms |
| --- | --- |
| 1 | COVID* or SARS-CoV-2 or Influenza or flu or myocardial infarction or heart failure or STI or STD or sexually transmitted* or infectious disease* or cardiovascular* or acute exacerbation or mycoplasma genitalium or chlamydia or gonorrhea or gonorrhoea or Group A strep* or pharyngitis or strep A or respiratory [abstract or title] |
| 2 | acute* or emergency [abstract or title] or (point of care* or point-of-care* or POCT or rapid test*) or (point of care and medical device*) or (point of care and diagnostic test*) or (point-of-care and medical device*) or point-of-care and diagnostic test* [abstract or title] |
| 3 | patient-report* or PRO or Self-report* or questionnaire* or clinician report* or clinRO [abstract or title] |
| 4 | **1 AND 2 AND 3** |

Abbreviations: POCT = point-of-care tests; COVID = coronavirus; ClinRO = clinician reported outcome; PRO = patient reported outcome; STI = sexually transmitted infection; STD = sexually transmitted disease

Table 4. Google scholar terms - Search 2: Development and / or validation of a PRO or clinician-outcome instrument evaluating POC tests

| No. | Search Terms |
| --- | --- |
| 1 | “patient reported outcome” “development” “point of care” |
| 2 | “patient reported outcome” “validation” “point of care” |
| 3 | “clinician reported outcome” “development” “point of care” |
| 4 | “clinician reported outcome” “validation” “point of care” |

Abbreviations: POC = point-of-care; COVID = coronavirus; PRO = patient reported outcome

## Supplementary Material B2: Inclusion / exclusion criteria (Search 1 and 2)

| Inclusion criteria |
| --- |
| - Abstract must report at least one of the following:   - The results of a primary or secondary (qualitative or quantitative) research study examining patient and / or healthcare professional experience, satisfaction, preference, perceptions of and / or knowledge or understanding of diagnostic tests   - Development and / or validation of a PRO and / or clinician-reported instrument assessing preference for or satisfaction with diagnostic tests   - Review of diagnostic test utilization in the USA, UK and / or Canada - Abstract must report research based in respiratory infections, cardiovascular diseases and / or sexually transmitted infections - Study population must be aged 18+ years - Abstract / full text must be in English language - Must be a journal article - Must be in a human population |
| Abbreviations: PRO = patient-reported outcome; USA = United States of America; UK = United Kingdom |

## Supplementary Material B3: Flow diagram of literature screening

**Identification**

Articles identified through database searches and supplementary searches in Google Scholar

**N = 392**

Existing material provided by one of the co-authors (SC)

**N = 21**

**Screening**

Abstracts screened

**N = 413**

Abstracts excluded *

**N = 373**

**Eligibility**

Full texts assessed for eligibility

**N = 40**

Full texts excluded

**N = 7**

**Included**

Studies included in qualitative synthesis

**N = 33**

*Abstracts were excluded based on the screening criteria detailed in Supplementary Material B2. Conference abstracts and duplicate publications were also excluded during this stage.

## Supplementary Material B4: Targeted literature review findings

Figure 1. Concepts impacting patient satisfaction

Note: Some papers reported multiple concepts, therefore, sub-concept counts may not match overall concept counts

Figure 2. Concepts impacting clinician satisfaction

Note: Some papers reported multiple concepts therefore sub-concept counts may not match overall concept counts

**Impact on patients’**

**diagnosis and**

**treatment (N=15)**

# Supplementary Material C – Data saturation: patient and clinician concept elicitation (CE) interviews

## Supplementary material C1: Saturation matrix for patient concept elicitation (CE) interviews

|  | Participant number | | | | | | | | | | | | | | | | | | | | | |
| --- | --- | --- | --- | --- | --- | --- | --- | --- | --- | --- | --- | --- | --- | --- | --- | --- | --- | --- | --- | --- | --- | --- |
|  | P1-UK | P2-CAN | P3-USA | P4-UK | P5-USA | P6-USA | P7-USA | P8-UK | P9-USA | P10-CAN | P11-CAN | P12-CAN | P13-USA | P14-CAN | P15-CAN | P16-USA | P17-USA | P18-CAN | P19-CAN | P20-CAN | TOTAL  S & P |  |
| **Patient concept** |  |  |  |  |  |  |  |  |  |  |  |  |  |  |  |  |  |  |  |  |  |  |
| Result wait time | S | P | P | P | P | S | S | S | S | P | P | P | P | P | P | S | P | P | P | S | 20 |  |
| Geographical location | P | S | S | P | P | P | P | S | S | P | P | P | P | P | P | P | P | P | P | S | 20 |  |
| Convenience | S | P | P | P | P | S | P | S | P | P | P | P | S | P | P | P | P | P | P | P | 20 |  |
| Administration time | P | S | S | P | P | P | S | S | S |  | P | P | P | S | S | P | P | P | S | P | 19 |  |
| Accuracy | P | S | P | P |  | P | P | P | P | P | P | P | P | P | P | P | P | P |  | S | 18 |  |
| Information provision | P | S | P | P | P | P | P | P | S | P | P | P | P |  |  | P | P | S | P | P | 18 |  |
| Accessibility | P | S | P | P |  | P | P | P | S | P | S | P | S | P |  | P | P | P | P | P | 18 |  |
| Method of result delivery | P | P | P | P |  | S | S | P | P | P | P |  | P | P |  | S | P | P | S | P | 17 |  |
| Hygiene | P | S | P | P | P | P | P | S |  | P | P | S | P |  | P |  |  | P | S | P | 16 |  |
| Sample collection method | S | S | P | P | S | S | P | P |  | P |  |  | P |  | P | S |  | P | S | P | 15 |  |
| Impact on clinical pathway | N | N | N | N | P | N | P | N | S | P | N | P | P |  |  | P | N | N | P | P | 9 |  |
| Emotional impact: result wait time | S |  | S | P | P | S |  | S |  | S |  |  | S |  |  | S |  |  |  |  | 9 |  |
| Ease of use | S | P |  |  |  | S |  | S | S |  |  | P |  |  |  |  | P |  |  | P | 8 |  |
| Appearance |  | P | P | P | P | P |  |  |  |  |  |  |  |  |  |  | P |  |  |  | 6 |  |
| Emotional impact: sample collection method |  |  |  |  | P | S | S |  |  |  |  |  |  |  |  | S | S |  |  |  | 5 |  |
| Note: Participant numbers listed in the order the interviews were conducted; S = Spontaneous mention (shaded cell is first spontaneous report of a concept); P=Probed mention; N = Participant reported no impact / not relevant; blank cells indicate that the concept was not (clearly) discussed  Abbreviations: UK = United Kingdom; USA = United States of America; CAN = Canada | | | | | | | | | | | | | | | | | | | | | | |

## Supplementary Material C2: Saturation matrix for clinician concept elicitation (CE) interviews

|  | Participant number | | | | | | | | | | | | | | | | | | | | |
| --- | --- | --- | --- | --- | --- | --- | --- | --- | --- | --- | --- | --- | --- | --- | --- | --- | --- | --- | --- | --- | --- |
|  | GP1-USA | IM2-USA | UC3-USA | IM4-USA | UC5-UK | UC6-UK | UC7-USA | IM8-USA | GP9-UK | GP10-UK | IM11-USA | NP12-CAN | GP13-UK | GP14-CAN | GP15-CAN | GP16-UK | GP17-CAN | GP18-CAN | GP19-CAN | GP20-UK | TOTAL S&P |
| **Clinician concept** |  |  |  |  |  |  |  |  |  |  |  |  |  |  |  |  |  |  |  |  |  |
| Interpretation of results | P | S | P | P | P | P | P | P | S | P | P | P | P | P | P | P | P | P | P | P | 20 |
| Ease of use | P | P | P | P | S | S | P | P | P | S | P | P | S | P | P | P | S | S | P | P | 20 |
| Result wait time | S | P | S | P | S | P | P | S | P | S | P | P | S | S |  | S | P | P | S | P | 19 |
| Impact on patients' clinical pathway | P | P | P | P | S | S | P | P | P | P | P | S |  | P | P | P | P | P | P | S | 19 |
| Convenience |  |  | P | P | S | S | P | P | S | S | S | S | P | P | P | P | P | P | P | P | 18 |
| Accuracy | S | S | P | P | S | P | S | S | S | S | P | P |  | S | S | P |  | P | P |  | 17 |
| Guidance and training | P |  | P | P | P | P | S | S |  | P | S | P | P |  | P | P |  | P | P | P | 16 |
| Administration time | P | P | S |  | S | P | P | P | S |  | P |  | S | P | P |  | S |  |  | P | 14 |
| Financial cost | P | P | P | P | S |  | S | P |  | P | P |  | N | P | P | N |  | P |  | P | 13 |
| Note: Participant numbers listed in the order the interviews were conducted; S = Spontaneous mention (shaded cell is first spontaneous report of a concept); P = Probed mention; N = Concept did not impact satisfaction; blank cells indicate that the concept was not (clearly) discussed  Abbreviations: GP = general practitioner; IM = internal medicine physician or internist; UC = urgent care or emergency doctor; UK = United Kingdom; USA = United States of America; CAN = Canada | | | | | | | | | | | | | | | | | | | | | |

# Supplementary Material D – Summary of clinical expert feedback on the first draft Patient Satisfaction with Diagnostic Test Questionnaire (PSDT-Q) and Clinician Satisfaction with Diagnostic Test Questionnaire (CSDT-Q)

## Supplementary Material D1: Summary of clinical expert feedback on the first draft Patient Satisfaction with Diagnostic Test Questionnaire (PSDT-Q)

| **PSDT-Q instruction / item** | **Clinical expert feedback** | **Summary of change (implemented in second draft)** |
| --- | --- | --- |
| Overall instructions | ‘Diagnostic test under study’ considered too complex for a patient-facing instrument | ‘Diagnostic test under study’ replaced with ‘the test’. |
| Item 1.3: Time to perform | ‘Complete’ considered ambiguous. Suggestion to ask patients assess satisfaction with time taken to ‘perform’ rather than ‘complete’ the test | ‘Complete’ changed to ‘perform’ |
| Item 1.5: Impact on emotional well-being: Result wait time | ‘Emotional well-being’ considered too complex for a patient-facing instrument | Item simplified to ask patient’s how they felt ‘emotionally’ while waiting for their result |
| Item 1.6: Method of result delivery | Include examples of result dissemination methods in the item description to facilitate understanding | Examples of result dissemination methods included in item description |
| Item 1.8: Impact on clinical pathway | Focus the item on the impact that a diagnostic has on the direction of a patient’s care rather than the impact on medical care more generally | Item updated to ask how the test impacted the direction of a patient’s medical care |
| Item 1.9: Accuracy | Patients are often not aware of the ‘accuracy’ of a test, confidence in accuracy is a more relevant concept to access | Item updated to assess confidence in accuracy |
| Item 1.9: Accuracy | Accuracy is a clinical term and not appropriate | ‘Accuracy’ replaced with ‘correct’ |
| Item 2.4: Hygiene | ‘Hygiene’ is not a common term and therefore not appropriate for a USA population | ‘Hygiene’ changed to ‘cleanliness’ |
| Item 2.5: Impact on emotional well-being: sample collection method | ‘Emotional well-being’ considered too complex for a patient-facing instrument. | Item simplified to ask patients how they felt ‘emotionally’ while waiting for their result |
| Response options: Satisfaction scale | Replace 7-point response scale with 5-point response scale | 5-point response scale implemented |

## Supplementary Material D2: Summary of clinical expert feedback on the first draft Clinician Satisfaction with Diagnostic Test Questionnaire (CSDT-Q)

|  | | |
| --- | --- | --- |
| **CSDT-Q instruction / item** | **Clinical expert feedback** | **Summary of change (implemented in second draft)** |
| Overall instructions | ‘Standard of care’ has an alternative meaning to what is intended (i.e., a legal term). Suggestion to replace with ‘standard care’. | ‘Standard of care’ replaced with ‘standard care’ |
| Overall instructions | ‘Diagnostic test under study’ is complex and reduces readability. Suggestion to simplify to ‘the test’ | Diagnostic test under study’ replaced with ‘the test’ |
| Item 1.7: Impact on workload / workflow | Satisfaction with test ‘efficiency’ considered unclear, the item explanation (i.e., ‘impact on workload / workflow’) was considered clearer | ‘Efficiency’ removed, and item explanation used as item stem |
| Item 2.1: Accuracy | Respondents may not be familiar with ‘sensitivity’, ‘specificity’ and ‘accuracy’ and / or consider the language unclear. Suggestion to assess clinician’s confidence in test results | Item revised to assess confidence in test result |
| Response options: Satisfaction scale | Response options need refining. Adding a ‘Neutral’ response option will allow respondent to indicate when they have genuine indifference regarding a concept | 7-point response scale with no neutral response option was refined to a 5-point response scale including a neutral response option |

# Supplementary Material E – Example revisions to the Patient Satisfaction with Diagnostic Test Questionnaire (PSDT-Q) and Clinician Satisfaction with Diagnostic Test Questionnaire (CSDT-Q) based on interview findings

## Supplementary Material E1: Example revisions to the Patient Satisfaction with Diagnostic Test Questionnaire (PSDT-Q) based on patient cognitive debriefing interviews

*PSDT-Q Example Revision 1: Item 1.3 (Time taken to perform test) wording*

**Original item wording:**

| How **satisfied or dissatisfied** are you with the **time taken to perform** the test?  *For example, the time taken to undergo the test.* |
| --- |

In round 1 of the patient cognitive debriefing interviews, most patients (n=4/5, 80%) understood this item. One patient did not understand if the item was assessing time taken to complete the test itself only, or if the time to access the test (i.e., to travel to the clinic) should also be considered. To address this, item wording was revised to specify time taken to ‘undergo’ the test and the example was amended to clarify that only the time taken to ‘collect the sample or measurement’ specifically should be considered.

**Revised item wording:**

| How **satisfied or dissatisfied** are you with the **time taken to undergo the test**?  *For example, the time taken to collect the sample or measurement.* |
| --- |

The item was well understood in round 2 and no issues were raised. As such, no further revisions were required.

*PSDT-Q Example Revision 2: Response Scale for Emotional Well-being Items*

**Original 5-point Likert response scale:**

| Very poor, Poor, Fair, Good, Excellent |
| --- |

In round 1 of the patient cognitive debriefing interviews, n=3/5 (60%) of patients found it difficult to select a response on the emotional impact items and indicated that the Likert scale response options were not appropriate for use. To address this, equivalent items assessing emotional well-being on a numeric response scale were developed (Section 3) prior to round 2.

**Revised response scale**:

| 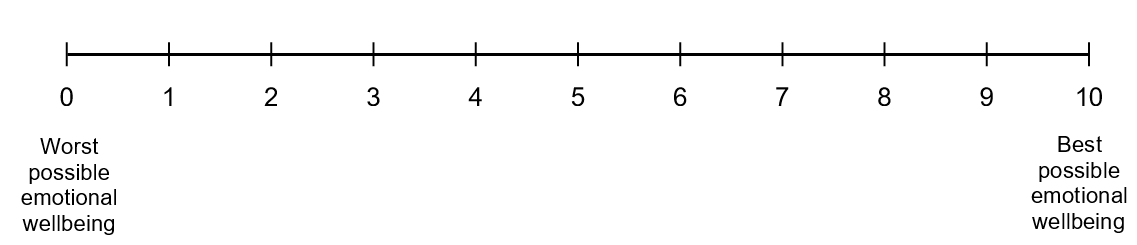 |
| --- |

In round 2, most patients preferred the items utilizing a numeric response scale vs a Likert scale (n=3/5, 60%) and the remaining patients were indifferent (n=2/5, 40%). The two items assessing emotional well-being on a Likert scale were subsequently removed from the PSDT-Q following round 2 in favor of the equivalent items utilizing a numeric response scale.

## Supplementary Material E2: Example revisions to the Clinician Satisfaction with Diagnostic Test Questionnaire (CSDT-Q) based on clinician cognitive debriefing interviews

*CSDT-Q Example Revision 1: Item 1 (Ease of use) wording*

**Original item wording:**

| The **ease of use**  *For example, how simple the test is to administer* |
| --- |

In the first round of clinician cognitive debriefing interviews, most clinicians (n=3/5, 60%) understood this item. However, one clinician discussed patients’ experiences and another discussed treatment in reference to this item, indicating a misunderstanding (n=2/5, 40%). The item was not revised, but additional probes were added to the round 2 interview guide to further explore this issue.

In round 2, most clinicians (n=3/5, 60%) understood the item but some (n=2/5, 40%) discussed other concepts, indicating misunderstanding. As this item was consistently misunderstood across rounds (n=4/10, 40%), item wording was revised following round 2 to clarify the intended concept for assessment.

**Revised item wording**:

| The **ease of use**  *For example, how simple the test is to administer or complete* |
| --- |

*CSDT-Q Example Revision 2: Item 8 (Instructions) wording*

**Original item wording:**

| The **instructions** on how to use  *For example, on how to collect and process a sample, set-up a machine or interpret the result* |
| --- |

In the first round of clinician cognitive debriefing interviews, only one clinician understood the original wording of this item. Lack of understanding was due to some clinicians (n=2/5, 40%) discussing instructions for patients and some (n=2/5, 40%) discussing the test process itself rather than instructions. The item was not revised, but further probes were added to the round 2 interview guide to explore this issue.

In round 2, some clinicians (n=2/5, 40%) understood the item. Those who did not understand this item discussed irrelevant concepts (test administration [n=2/3, 66%]; patient instructions [n=1/3, 33%]). Based on the consistency of misunderstanding across rounds (n=4/8, 50%) the item was revised following round 2 to explicitly state that ‘instructions for you or your staff’ should be considered when responding.

**Revised item wording**:

| The **instructions** on how to use  *For example, instructions for you or your staff on how to collect and process a sample, set-up a machine or interpret the result* |
| --- |

# Supplementary Material F – Patient Satisfaction with Diagnostic Test Questionnaire (PSDT-Q) and Clinician Satisfaction with Diagnostic Test Questionnaire (CSDT-Q) item tracking matrices

## Supplementary Material F1: Patient Satisfaction with Diagnostic Test Questionnaire (PSDT-Q) item tracking matrix: Summary of participant feedback that informed evidence-based revisions

| PSDT-Q content | Round 1 feedback | Revision | Round 2 feedback | Revision |
| --- | --- | --- | --- | --- |
| *Section 1 – Satisfaction with diagnostic test process* | | | | |
| Item 1.2: Ease of completion | n=2/5 did not clearly understand the item,  but n=5/5 found the term ‘undergo the test’ clear | ‘Undergo the test’ wording implemented and item re-phrased to improve clarity | n=4/5 did not clearly understand the item | Item wording reverted due to better performance in Round 1 |
| Item 1.3: Time taken to perform | P4-UK-STI-CD did not understand if the item related to the time taken to complete or access the test | ‘Test’ bolded and wording revised to clarify item assesses time taken to complete the test only | n=5/5 understood and n=3/5 considered relevant | No changes |
| Item 1.5: Method of result delivery | n=2/5 suggested revising wording to ‘method of receiving results’ to improve clarity | ‘Method of receiving results’ wording implemented | n=4/5 understood and considered relevant | No changes |
| Item 1.6: Ease of understanding | n=2/5 did not consider relevant for inclusion as did not receive a test result | ‘I did not receive my test result’ response option added (update also applied to items 1.5 and 1.7) | n=5/5 understood and considered relevant | No changes |
| Item 1.8: Impact on clinical pathway | n=5/5 understood and n=3/5 considered relevant | Wording revised to assess satisfaction with how the 'test' impacted clinical pathway, rather than the 'test result’ (as not all participants received results; see item 1.6 feedback) | n=4/5 understood, and P6-USA-RESP-CD considered relevant | Item wording reverted due to better performance in Round 1, and ‘Not applicable’ response option added |
| *Section 2 – Satisfaction with sample or measurement collection* | | | | |
| Item 2.1: Comfort | N/A | Item added during Round 2 (debriefed by N=3) | n=3/3 understood and n=2/3 considered relevant | Item was well understood so moved to the start of the section, examples of ‘sample and measurement’ were added to the description, and a grammatical update was made |
| Item 2.3: Type of sample or measurement | P3-USA-CVD-CD did not consider an echocardiogram to be a ‘sample’ and suggested the terms ‘measurement’ or ‘reading’. P5-CAN-RESP-CD was diagnosed via clinical opinion, so suggested including the term ‘procedure’ | Wording updated to ‘sample or measurement’ (update also applied to items 1.3, 2.2, 2.4 and 2.5). More examples of diagnostic evaluations were added to the description | n=2/5 understood and considered relevant | Item retained as concept identified as key in the targeted literature review and did not receive notable feedback from clinical experts |
| *Section 3 – Emotional well-being during diagnostic test process* | | | | |
| Item 3.1: Overall | N/A | Item added following Round 1 | n=3/5 understood.  P10-UK-CVD-CD referred to their emotional state when the test was being performed only | ‘Overall’ term was added (mid-round) and bolded. Item moved to the start of section so the global item preceded concept-specific items (update also applied to item 4.1) |
| Response option:  5-point Likert, Emotional well-being scale | n=2/3 asked found the response options inappropriate,  n=2/5 suggested including response options relating to specific emotions: P1-UK-STI-CD suggested including an open-text response box and P5-CAN-RESP-CD suggested including a ‘not applicable’ response option | Section 3 added including emotional well-being items utilizing a 0-10 numeric response scale (NRS) | n=3/5 preferred the NRS and n=2/5 were indifferent; no participants expressed a preference for the Likert scale | Emotional well-being items utilizing a 5-point Likert scale were removed, items utilizing a 0-10 NRS were retained |
| *Section 4 – Preferences for diagnostic tests [Optional add-on module]* | | | | |
| Section header | n=4/5 understood and n=3/3 asked found it easy to respond to | No change | n=2/5 did not understand the need to compare two tests | Wording revised to clarify two diagnostic tests are being compared, and  placeholders for test names added |
| Response option (item 4.1):  5-point Likert, Preference scale | n=2/5 found it easy to select a response on this scale. P2-USA-CVD-CD found some response options difficult to differentiate, P1-UK-STI-CD found term ‘preference’ too vague | Grammatical updates made to response scales for consistency (update also applied to the response options for items 4.2 and 4.3) | n=3/4 asked found it easy to select a response on this scale | No changes |
| **Abbreviations:** N = Number of participants; PSDT-Q = Patient Satisfaction with Diagnostic Test Questionnaire | | | | |

## Supplementary Material F2: Clinician Satisfaction with Diagnostic Test Questionnaire (CSDT-Q) item tracking matrix: Summary of participant feedback that informed evidence-based revisions

| CSDT-Q content | Round 1 feedback | Revision made | Round 2 feedback | Revision made |
| --- | --- | --- | --- | --- |
| *Section 1 – Satisfaction with the ‘test’ and ‘standard care’* | | | | |
| Section header | n=3/5 considered the instructions clear | No changes | n=5/5 found the instructions clear, but  GP9-CAN-CVD-CD discussed satisfaction from the patient perspective on several items and responded to items which were not applicable to them | Instructions outlining when to use the ‘not applicable’ option were bolded to clarify when it should be utilized |
| Item 1: Ease-of-use | n=3/5 understood and considered relevant, n=2/5 did not clearly understand | No changes | n=3/5 understood and considered relevant, n=2/5 did not clearly understand as discussed other concepts as well as ease-of-use | Wording revised (‘administer’ revised to ‘administer or complete’) to improve clarity |
| Item 8: Guidance and training | n=2/5 discussed satisfaction with the test process itself, n=2/5 referenced the patient perspective | No changes, but interview guide revised to explore further | n=2/5 discussed administering the test as well as satisfaction with the instructions, and GP9-CAN-CVD-CD referenced the patient perspective | Wording revised to explicitly state that instructions for clinicians only should be considered, and the example was revised to clarify focus on satisfaction with instructions only |

| **Abbreviations:** N = Number of participants; CSDT-Q = Clinician Satisfaction with Diagnostic Test Questionnaire |
| --- |
